# Distinguishing caffeine expectancy from pharmacological effects: a comparative review of experimental designs with future directions

**DOI:** 10.3389/fphys.2026.1865993

**Published:** 2026-06-11

**Authors:** Slaheddine Delleli, Javier Diaz-Lara, Hamdi Chtourou

**Affiliations:** 1 High Institute of Sport and Physical Education of Sfax, University of Sfax, Sfax, Tunisia; 2 Research Unit: Physical Activity, Sport and Health, National Observatory of Sport, Tunis, Tunisia; 3 Performance and Sport Rehabilitation Laboratory, Faculty of Sports Sciences, University of Castilla-La Mancha, Toledo, Spain

**Keywords:** Belief, caffeine, deception, ergogenic, performance, synergy

## Abstract

Caffeine (CAF) is among the most widely consumed bioactive substances and is recognized for its ergogenic benefits. Yet, separating its expectancy-driven effects from pharmacological effects remains challenging. This review compares the randomized double-blind placebo-controlled crossover, deceptive placebo-balanced, and double-deceptive placebo-balanced crossover designs commonly used in CAF studies. While the double-blind crossover design effectively controls individual variability, it cannot directly measure expectancy. In contrast, placebo-balanced crossover designs incorporating deception provide the most comprehensive framework when the aim is to separate pharmacological and expectancy-related effects, allowing a more precise interpretation of caffeine’s mechanisms. Differences in studies designs may partially explain inconsistencies in the literature, as expectancy-related components are not equally controlled across experimental approaches.

## 1 Introduction

Caffeine (CAF) is the most commonly consumed psychostimulant recognized for its ability to enhance alertness, pain tolerance, and physical performance (Reddy et al., 2024). Its ubiquity across daily routines has led to extensive research into its pharmacological and psychological effects, particularly within physical exercise and sports settings (Grgic et al., 2020; Sharma et al., 2023). Although the ergogenic benefits of CAF are well-documented, the precise interaction between its pharmacological mechanisms and expectancy-related effects remains incompletely understood (Beedie, 2010; Hurst et al., 2020). Mechanistically, CAF acts primarily as an adenosine receptor antagonist (Nehlig, 2018; Sharma et al., 2023). Through this antagonism CAF may enhance fat oxidation, reduce the perception of fatigue, increase calcium ion release in skeletal muscle, and improve neuromuscular transmission and central nervous system excitability (Tarnopolsky, 2008; Davis and Green, 2009; Van Schaik et al., 2021). However, inconsistent findings across studies suggest that these biochemical effects alone cannot fully explain CAF ‘s performance-enhancing outcomes (Beedie et al., 2015; Shabir et al., 2018; Pickering, 2019). Psychological expectancy, defined as the belief that one has consumed CAF, can independently modify physiological and perceptive responses (Duncan et al., 2009; Beedie, 2010).

Several investigations have demonstrated that expectancy effects can produce measurable improvements in performance and mood, even without actual CAF intake (Beedie et al., 2006; Foad et al., 2008; Dawkins et al., 2011; Del Coso et al., 2026). Such effects are mediated through cognitive and motivational pathways, potentially involving dopaminergic activation similar to that induced by CAF itself (Kaasinen et al., 2004; Shabir et al., 2018). Consequently, CAF’s ergogenicity should be understood as a dual phenomenon both pharmacological and expected in nature (Beedie et al., 2018). To address this complexity, researchers (Foad et al., 2008; Tallis et al., 2016; Shabir et al., 2019; Hurst et al., 2020; Bezuglov et al., 2025; Soto et al., 2025; Del Coso et al., 2026; Soares et al., 2026) have adopted increasingly refined experimental frameworks. The Randomized double-blind placebo controlled cross-over design (RDBPCCD) is considered the gold standard for quantifying pharmacological effects while minimizing bias (Kaptchuk, 2001). Each participant receives both CAF and placebo (PL) in random order, separated by a washout period, thereby reducing interindividual variability (Grgic et al., 2020). However, because expectations are neutralized by blinding-control, this design does not allow the assessment of expectancy effects (Beedie et al., 2015). Importantly, recent evidence indicates that expectancy effects may vary depending on the outcome measured. While performance-related outcomes often show modest or inconsistent effects, physiological variables such as substrate oxidation may be more sensitive to expectancy manipulations (Gutiérrez-Hellín et al., 2021).

To assess CAF expectancy-driven effects, scientists introduced the deceptive placebo-balanced design (DPBD), which manipulates participants’ expectations by deceiving them about whether CAF or PL was administered (Lotshaw et al., 1996; Enck et al., 2011). This approach allows the investigation of expectancy-driven effects in isolation (Filip-Stachnik et al., 2020; Valero et al., 2026). However, it does not allow a clear separation between pharmacological and expectancy effects. To examine whether pharmacological and expectancy effects interact, more complex designs such as double-deception or double-dissociation placebo-balanced crossover designs (DDPBCD) have been implemented (Foad et al., 2008; Hurst et al., 2020; Soares et al., 2026). These designs have revealed complex and sometimes counterintuitive interactions (Del Coso et al., 2026). Despite being resource-intensive and ethically demanding, these protocols provide the most comprehensive framework when the aim is to separate pharmacological and expectancy-related effects (Hurst et al., 2020; Del Coso et al., 2026). Collectively, these methodological approaches have improved our understanding of CAF’s multifaceted effects. However, the choice of the appropriate design remains unclear in CAF studies. Differences in experimental design may partly explain inconsistencies in the literature, as expectancy effects are not always controlled. Therefore, the present review compares the randomized double-blind placebo-controlled crossover, deceptive placebo-balanced, and double-deception placebo-balanced crossover designs, highlighting their strengths, limitations, and implications for future research in sport and exercise science.

## 2 Search strategy and studies selection

PubMed and Google Scholar databases were searched without filters or time and language restriction to reduce the risk of missing relevant studies (Rico-González et al., 2022). The search syntax included the following keywords: Caffeine, placebo-balanced, deceptive balanced, double-deceptive, double-blind randomized cross-over, expectancy, belief, pharmacological effects, design, trial. Appropriate Boolean connectors (AND, OR) were utilised appropriately to connect the various keywords. An additional search was performed by screening the reference lists of the included studies and related review papers. Studies were selected after screening titles, abstracts and keywords when the following eligibility criteria were met: i) being CAF research regardless of dosage, ii) presenting one of the compared designs, iii) being a placebo-controlled study, and iv) assessing CAF ergogenic potential on physical, physiological, perceptive, or cognitive responses in healthy population. The search result was imported using the software “Endnote 20” (Camelot UK Bidco Limited-Clarivate, UK). Afterwards, relevant studies were grouped based on their design.

## 3 Caffeine pharmacological effects

CAF exerts its primary physiological action through non-selective antagonism of adenosine receptors, mainly A_1_ and A_2_A subtypes, which prevents adenosine from binding and exerting its inhibitory influence on neuronal activity (Fredholm et al., 1999; Davis et al., 2003). By blocking these receptors, CAF reduces the perception of fatigue and promotes increased neuronal firing in the central nervous system, leading to elevated arousal, vigilance, and sustained attention (Nehlig et al., 1992; Davis and Green, 2009; Nehlig, 2018). This adenosine blockade enhances dopaminergic and noradrenergic signaling, particularly in the striatum and prefrontal cortex, which underlies improvements in psychomotor performance (Kaasinen et al., 2004; Ferré, 2016; Pires et al., 2018). CAF’s stimulatory effect also extends to the sympathetic nervous system, triggering catecholamine release; especially epinephrine and norepinephrine, resulting in increased heart rate, blood pressure, and energy expenditure (Spriet, 2014; Barcelos et al., 2020). From a metabolic standpoint, CAF promotes lipolysis and glycogen sparing, increasing the mobilization of free fatty acids during exercise (Graham, 2001). This shift toward fat oxidation contributes to improved endurance capacity and delayed onset of fatigue. CAF also augments intracellular calcium release from the sarcoplasmic reticulum in skeletal muscle, facilitating excitation-contraction coupling and muscular force output (Tarnopolsky, 2008; Grgic et al., 2020). Moreover, its thermogenic effects mediated via adipose tissue thermogenesis activation further enhance energy expenditure, overall metabolism rate and energy homeostasis (Van Schaik et al., 2021; Soto et al., 2025). While these physiological mechanisms are beneficial, the ergogenic effects of CAF follow a dose-dependent, inverted-U pattern. In fact, moderate doses (3–6 mg·kg^−1^) reliably enhance endurance, attention, and strength, whereas higher intakes (>9 mg·kg^−1^) can provoke anxiety, tachycardia, or gastrointestinal discomfort (de Souza et al., 2022; Saimaiti et al., 2023). Chronic overconsumption may impair sleep, elevate blood pressure, and induce tolerance due to receptor upregulation and altered adenosine sensitivity (Lee et al., 2026). Neurocognitively, CAF improves executive function, working memory, and reaction speed, partly by enhancing dopamine release in mesolimbic pathways (Kaasinen et al., 2004; Unsal and Sanlier, 2025). These neural effects contribute to perceived improvements in motivation and effort regulation, which are often paralleled or even mimicked by expectancy-driven mechanisms (Beedie et al., 2006; Dubljanin et al., 2026). Thus, while CAF’s pharmacological action clearly enhances both physical and cognitive performance, distinguishing its true pharmacological effects from psychologically mediated expectancy responses remains a central challenge in experimental research.

## 4 Caffeine expectancy-driven effects

Psychological and physiological responses arising from a person’s belief about a treatment rather than the treatment itself play a critical role in CAF studies (Colagiuri, 2010; Beedie et al., 2018). These effects are conceptually rooted in classical conditioning and cognitive appraisal, whereby prior experiences and contextual cues generate anticipatory neurobiological responses (Colloca and Benedetti, 2006; Dubljanin et al., 2026). Within this framework, CAF expectancy can influence both perceived exertion and actual physiological performance, sometimes producing outcomes comparable to genuine pharmacological stimulation (Beedie et al., 2006; Hurst et al., 2020).

The DPBD and its modern adaptations (i.e., DDPBCD) provide the clearest methodological means of quantifying these effects (George et al., 2012). Empirical evidence demonstrates that expectancy alone can enhance exercise performance, mood, and attentional control. However, expectancy-only designs allow the isolation of belief-related effects but do not permit direct comparison with pharmacological effects within the same experimental framework (Dawkins et al., 2011; Shabir et al., 2019). Neuroimaging studies have shown that CAF expectancy activates dopaminergic circuits in the striatum and anterior cingulate cortex, similar to true CAF ingestion (Kaasinen et al., 2004; Cauli and Morelli, 2005). For instance, Pires et al. (2018) reported that perceiving PL as CAF may boost a cognitive top-down effect induced corticospinal excitability resulting in similar cerebral and motor performance responses between CAF and PL expected as CAF. Such evidence underscores that expectancy is not a mere psychological artifact but engages genuine neurophysiological mechanisms capable of modulating behavior. However, expectancy effects are not universally positive or predictable. Some investigations reveal that believing one has consumed CAF can lead to no change or even impaired performance in highly trained athletes who rely on precise pacing or arousal control (Tallis et al., 2016; Filip-Stachnik et al., 2020). Negative expectancy believing that a PL is CAF or vice versa can elicit outcomes opposite to those anticipated, illustrating a nocebo-like dimension of CAF studies (Beedie et al., 2007; Foad et al., 2008; Chhabra and Szabo, 2024). These divergent findings suggest that expectancy interacts dynamically with contextual factors such as habitual CAF intake, personality traits, prior experiences, and task type (Colloca and Benedetti, 2006; Beedie et al., 2015; Pickering and Kiely, 2018).

The introduction of the DDPBCD has further advanced understanding of expectancy phenomena and its interaction with pharmacological effects (Bezuglov et al., 2025; Del Coso et al., 2026). In this paradigm, participants are independently manipulated in terms of what they are told (i.e., belief) and what they actually receive (i.e., pharmacological substance). Such manipulation results in four conditions: told CAF/given CAF, told CAF/given PL, told PL/given CAF, and told PL/given PL (Lotshaw et al., 1996; Enck et al., 2011). By exposing each participant to all four told/given conditions, this within-subject approach isolates pharmacological and psychological components with greater precision (Foad et al., 2008; Bjørkedal and Flaten, 2011). Findings from these studies consistently highlight significant belief × pharmacology interactions, confirming that performance benefits are not purely biochemical but shaped by conscious expectation (Beedie, 2010; Bjørkedal and Flaten, 2011; Del Coso et al., 2026). Overall, expectancy effects in CAF studies reflect a complex interplay between cognition, emotion, and neurobiology. They demonstrate that the perceived belief in CAF ingestion can modulate central drive, effort regulation, and motor output, thereby challenging the assumption that CAF’s ergogenic benefits are solely pharmacological (Del Coso et al., 2026). Using the appropriate design to accurately measure these effects is essential for valid interpretation of experimental findings and for designing ethically sound, ecologically valid studies.

## 5 Strengths and limitations of caffeine’ designs

As presented in **Table 1**, each of the compared CAF’ research design has specific strength and limitations.

**Table 1:** Comparative analysis of experimental designs used to examine caffeine’s pharmacological and expectancy effects.

Design feature	Randomized double-blind placebo controlled cross-over design	Expectancy-only deceptive design (no active treatment)	Deceptive partial crossover design	Double-deception (Full factorial crossover) design
Typical structure	PL vs. CAF, both under blinded conditions	Participants receive PL, but expectancy is manipulated through information/deception	PL, CAF, and a deceptive condition (e.g., informed CAF)	Told PL/received PL; told PL/received CAF; told CAF/received PL; told CAF/received CAF
Expectancy Manipulation	No (only indirectly controlled by blinding)	Yes	Yes	Yes
Can estimate pharmacological effects	Yes	No	Yes, but not fully separated from expectancy effects	Yes
Can estimate the expectancy effect	No	Yes	Yes, but not fully separated from expectancy effects	Yes
Can estimate inter-action	No	No	No	Yes
Main strengths	Strong internal validity for estimating pharmacological effects; widely used; relatively simple design	Useful to isolate belief-related effects in the absence of pharmacological input	Useful to show that expectancy can produce measurable effects and to compare them with actual CAF	Most complete design for separating pharmacological, expectancy, and interaction effects within the same experiment
Main limitations	Does not experimentally isolate expectancy effects	Cannot compare expectancy with actual CAF within the same full framework; no pharmacological arm. Not a fully standardized design category in the literature	Does not include the full 2 × 2 crossing of information and substance; cannot fully disentangle interaction effects	More complex, resource-intensive, and ethically demanding; manipulation checks are especially important
Example	(Duncan et al., 2009; Diaz-Lara et al., 2016; Giraldez-Costas et al., 2022)	(Beedie et al., 2006; Filip-Stachnik et al., 2020; Ortiz-Sánchez et al., 2024; Valero et al., 2026)	(Gutiérrez-Hellín et al., 2021)	(Foad et al., 2008; Tallis et al., 2016; Shabir et al., 2019; Hurst et al., 2020; Bezuglov et al., 2025; Soto et al., 2025; Del Coso et al., 2026; Soares et al., 2026)

CAF, caffeine; PL, placebo.

### 5.1 Randomized double-blind placebo controlled cross-over design

The RDBPCCD is considered as the gold standard for investigating pharmacological interventions such as CAF. In this design, each participant receives both the active treatment (CAF) and the PL in a randomized order, with a sufficient washout period between sessions to eliminate residual drug effects (Ernst and Resch, 1995). This within-subject structure enables participants to serve as their own controls, thereby minimizing interindividual variability and increasing statistical power (Atkinson and Batterham, 2015; Barnes, 2024). Because each participant undergoes both experimental conditions, confounding factors such as genetics, habitual CAF intake, training status, and motivational differences are inherently controlled (Guest et al., 2021). This structure is particularly advantageous in exercise physiology, where interindividual variation in CAF metabolism (e.g., CYP1A2 and ADORA2A genotypes) can significantly affect ergogenic outcomes (Nehlig, 2018). Randomization and double-blinding reduce experimenter and participant bias, allowing for a more accurate estimation of CAF’s true pharmacological influence on performance and cognition (Saunders et al., 2017). Moreover, crossover designs are statistically efficient, often requiring smaller samples than parallel-group trials to detect the same effect size (Jones and Kenward, 2003).

However, despite these strengths, the double-blind crossover approach has several methodological limitations. First, while blinding aims to minimize expectancy, it also prevents direct measurement of belief-related or psychological influences (Beedie et al., 2015). Consequently, the design may underestimate the total performance effect when expectancy contributes substantially to outcomes (Hurst et al., 2020). Second, carryover effects in which the physiological or psychological effects of CAF persist into the subsequent session can confound results if the washout period is insufficient (Colloca and Benedetti, 2006). Even moderate CAF doses can affect sleep quality, arousal, and mood for several hours or days, potentially altering baseline conditions before the next session (Juliano et al., 2019). Another concern involves learning and habituation effects (Shabir et al., 2019). Repeated testing in crossover trials can produce training-related improvements unrelated to the treatment itself, particularly in performance tasks requiring skill or pacing strategy (Ernst and Resch, 1995). Habituation to CAF both physiological and perceptual can also reduce responsiveness over time, complicating within-subject comparisons (Sökmen et al., 2008). Moreover, the assumption of perfect blinding may not hold; participants often correctly guess whether they received CAF due to recognizable physiological cues such as increased heart rate or alertness (Duncan et al., 2009; Hurst et al., 2020). Such blinding failure introduces expectancy effects even in ostensibly “blind” designs. While the RDBPCCD remains widely accepted framework for isolating CAF ‘s pharmacological effects, this design alone cannot fully capture the psychological and contextual components of CAF’s ergogenic effects (Colagiuri, 2010). Importantly, this limitation may contribute to inconsistencies observed across studies, as expectancy-related effects are not equally controlled across experimental approaches.

### 5.2 Deceptive placebo-balanced design

The DPBD was developed to explore expectancy-driven effects; how belief alone shapes behavioral and physiological outcomes (Filip-Stachnik et al., 2020; Gutiérrez-Hellín et al., 2021). When implemented without an active CAF treatment (i.e., expectancy-only), it isolates the pure effect of believing CAF has been consumed (Beedie et al., 2006). However, these expectancy-only approaches do not allow direct comparison with pharmacological effects within the same experimental framework. Such designs have revealed that mere belief in CAF consumption can improve mood, alertness, and pacing even when participants receive an inert PL (Beedie et al., 2006; Duncan et al., 2009). The DPBD is especially valuable in CAF studies because expectancy effects can, in some cases, be comparable to pharmacological effects (Beedie et al., 2006; Hurst et al., 2020). When participants believe they have ingested CAF, improvements are often observed in endurance, strength, power, reaction time, and subjective vitality even when CAF is absent (Duncan et al., 2009; Dawkins et al., 2011; Valero et al., 2026). Such findings emphasize that belief, motivation, and attentional focus can meaningfully modulate physiological and psychological outcomes in sport and exercise contexts (Benedetti and Colloca, 2003; Soares et al., 2026). From a neurobiological perspective, expectancy manipulations influence cortical activation pathways, eliciting genuine changes in perceived effort, pain tolerance, and reward processing (Kaasinen et al., 2004). These findings support the concept that expectancy effects are physiologically real and not merely psychological artifacts (Ortiz-Sánchez et al., 2024). However, expectancy-only studies lack the ability to assess the true magnitude of pharmacological versus expected effects, as they omit active treatment arms. Ethically, deception remains a challenge; researchers must justify false information and ensure post-study debriefing (Waring, 2008). Nonetheless, DPBD studies contribute uniquely to understanding how cognitive beliefs and social context influence ergogenic responses, particularly in sports where supplement expectations are widespread (Dawkins et al., 2011; Shabir et al., 2018).

### 5.3 Double-deception placebo-balanced crossover design

Integrating double-deception or hybrid designs that combine the pharmacological control of crossover studies with the belief manipulation of deceptive placebo-balanced protocols has been proposed to yield a more comprehensive understanding of CAF’s dual mechanisms (Dawkins et al., 2011; George et al., 2012). The DDPBCD is a methodological framework specifically developed to disentangle the pharmacological and expectancy-driven components of a substance’s effect (Enck et al., 2011; Hurst et al., 2020). In this design, participant expectations are deliberately manipulated by crossing what participants are told (belief) with what they actually receive (treatment) (Lotshaw et al., 1996). This 2 × 2 factorial structure enables the separation of the pure pharmacological effect (CAF vs. PL), the pure expectancy effect (belief vs. disbelief), and the interaction effect between pharmacology and cognition (Foad et al., 2008; Beedie, 2010). Such designs provide a more comprehensive framework to examine how belief and pharmacological effects interact, although the magnitude and direction of these effects may depend on the outcome measured (Del Coso et al., 2026). However, DDPBCD studies require larger sample size, complex logistics, and careful ethical consideration due to deliberate deception (Waring, 2008; Dubljanin et al., 2026). They also rely heavily on effective manipulation checks to confirm participants’ beliefs (Ortiz-Sánchez et al., 2024). Nonetheless, they remain the most comprehensive approach when true aim is separate to compare pharmacological and expectancy-related effects.

## 6 Discussion

Understanding CAF’s dual influence as well as its pharmacological action and expectancy-driven effects requires methodological designs capable of disentangling these intertwined mechanisms. The three dominant paradigms in this field offer complementary yet distinct insights, each with specific advantages and limitations. One key point is that differences in studies designs may explain some of the inconsistent findings, as expectancy effects are not equally accounted for across investigations.

The RDBPCCD remains the most commonly employed in CAF studies due to its efficiency, internal validity, and statistical power (Kaptchuk, 2001). By having each participant serve as their own control, this design allows a robust control of confounding variables to minimize interindividual variability, making it ideal for quantifying true pharmacological responses (Atkinson and Batterham, 2015). By ensuring that neither participants nor investigators are aware of the treatment condition, this minimizes both experimenter bias and expectancy-related confounds (Tallis et al., 2022). However, its core strength (i.e., blinding) also constitutes its primary limitation. It effectively neutralizes expectancy, preventing direct assessment of psychological belief effects that may mediate performance outcomes (Colagiuri, 2010; Beedie et al., 2015). Furthermore, subtle physiological cues (i.e., side effects) such as increased heart rate or alertness may compromise blinding, introducing unintended expectancy influences (Bjørkedal and Flaten, 2011; Hurst et al., 2020). The distinct bitter taste and physiological cues of CAF can jeopardize true blinding where participants are able to identify that they have consumed CAF, a phenomenon known as “pseudo-blinding” (Pickering, 2019). This awareness may unintentionally reintroduce expectancy bias, thereby compromising the validity of double-blind methodologies (Colagiuri, 2010). Consequently, researchers have emphasized the need for improved PL formulations that more effectively mimic CAF’s sensory and physiological characteristics, maintaining blinding integrity (Pickering, 2019; Tallis et al., 2022).

A deceptive experimental design aims to manipulate participants’ beliefs about the substance they are receiving, often by deliberately misrepresenting the intervention to isolate the potential effect of expectation (Beedie et al., 2006). This form of expectancy manipulation allows researchers to evaluate how cognitive beliefs, rather than pharmacological action, influences physiological and performance outcomes (Colagiuri, 2010). In CAF studies, such deception-based protocols particularly DPBD and DDPBCD have provided valuable insights into the interaction between mind and physiology, revealing that belief alone can elicit ergogenic or affective benefits comparable to those produced by CAF itself (Beedie et al., 2006; Duncan et al., 2009).

The DPBD provides a conceptual solution by explicitly manipulating expectancy through controlled deception (Shabir et al., 2019). Participants may be told they have ingested CAF or PL, regardless of the actual treatment, enabling researchers to separate belief-driven effects from pharmacological effects. Expectancy-only variants of this design where all participants receive PL are particularly useful in assessing the magnitude of psychological influences independent of pharmacological input (Duncan et al., 2009). These designs, however, face ethical challenges due to intentional deception, as well as logistical demands such as multiple participant briefings, manipulation checks, and thorough debriefings to maintain ethical transparency (Waring, 2008). Nevertheless, DPBD studies have demonstrated that expectancy alone can influence performance, highlighting psychological belief as an active determinant (Saunders et al., 2017). Verifying participants’ beliefs is essential, yet challenging, and the absence of such check’s limits interpretation of expectancy effects. While the psychometrically validated Stanford Expectations of Treatment Scale (SETS) (Somogyi et al., 2025) and the Brief-CAF Expectancy Questionnaire (B-CaffEQ) (Mendes GF et al., 2021) could be used to subjectively assess the initial CAF expected effects, they are not useful to check consumer belief after manipulating expectancy.

Building on the aforementioned designs, the DDPBCD represents the most comprehensive approach when the aim is to separate pharmacological and expectancy effects. It systematically exposes participants to all four combinations of belief and treatment (Del Coso et al., 2026). This factorial structure allows simultaneous estimation of main effects (belief vs. pharmacology) and interaction effects (belief × pharmacology) within the same individuals. Findings from such designs reveal complex interactions where both CAF and expectancy independently improve performance, but their combined influence is not always additive and may depend on the outcome measured (Del Coso et al., 2026) or even non-additive depending on context (Bjørkedal and Flaten, 2011). While offering unparalleled analytical precision, double-deception designs are resource-intensive, requiring multiple visits, extensive participant management, and rigorous ethical justification due to repeated deception (Benedetti and Colloca, 2003).

## 7 Future directions

While the RDBPCCD and DPBD represent a continuum of experimental control and ecological realism of CAF pharmacological and expectancy only effects, using an hybridize approaches combining robust pharmacological blinding with embedded expectancy manipulations could be the appropriate framework to assess the possible synergistic effects of CAF actions. Such innovation would enable CAF studies to move beyond pharmacological determinism and toward a holistic understanding of how belief, neurobiology, and context jointly influence human performance. Future studies could combine traditional pharmacological designs with approaches that also take expectancy into account, as this may help better understand the caffeine actions in real settings. These types of designs could help clarify how much of the effect is due to the substance itself and how much is related to belief, as well as whether both interact. Such integration would enable researchers to map how expectancy translates into measurable brain and behavioral changes during exercise. It is also important to treat expectancy as a relevant variable, not just as noise. In this context, checking whether participants believed what they were told becomes essential to properly interpret the results. In addition, future work should incorporate open science practices and ethical transparency in deception-based designs. This includes preregistration of protocols, and clear justification of methodological choices to ensure participant welfare and scientific integrity (Benedetti and Colloca, 2003). By combining methodological innovation with ethical rigor, CAF studies can progress toward a holistic understanding of how pharmacological, psychological, and contextual factors converge to influence performance and cognition. Researchers must ensure that deception is scientifically justified, risk-free, and followed by transparent debriefing to maintain trust and ethical integrity (Beedie et al., 2015). Moreover, implementing four experimental conditions requires greater participant commitment and careful counterbalancing to prevent order effects or expectancy learning across sessions (Saunders et al., 2017). Equally critical are manipulation checks, which confirm whether the expectancy manipulation was successful. Without such verification, the validity of expectancy-related inferences is compromised. Manipulation checks typically involve post-trial questionnaires asking participants to report whether they believed they had ingested CAF or PL and how confident they were in that belief. These measures are essential to interpreting observed effects as genuinely expectancy-driven rather than coincidental or uncontrolled. Although this review focuses on caffeine, these same ideas could likely apply to other ergogenic aids such as creatine, nitrate or beta-alanine, where expectations may also play a role.

## 8 Conclusion

Understanding CAF’s ergogenic effects requires both methodological sophistication and conceptual clarity. Studies to date demonstrate that CAF’s influence on performance is not solely biochemical but also profoundly shaped by cognitive, motivational, and contextual factors. CAF ergogenic potential has been assessed using different research frameworks. The randomized double-blind placebo controlled cross-over design was the most effective framework for isolating CAF’s pharmacological efficacy, ensuring rigorous internal validity and minimizing bias. In contrast, the placebo-balanced design provides unique insight into the psychological and expectancy-driven components of performance enhancement. These paradigms reveal that CAF’s ergogenic effects are not independent of belief-related influences. Looking forward, understanding how pharmacological and expectancy-related effects interact will require integrating these paradigms. Such approaches enable researchers to: i) quantify independent and interactive effects of belief and pharmacology on performance related outcomes, ii) control for interindividual variability, including habituation, genotype, and CAF sensitivity, and may help explain part of the variability reported in the literature due to differences in studies designs iii) and enhance ecological validity by replicating real-world consumption and motivational contexts. Overall, improving studies designs will be key to better understanding how CAF influences performance, taking into account both physiological and expectancy-related factors.
